# Peer Verbal Encouragement Enhances Offensive Performance Indicators in Handball Small-Sided Games

**DOI:** 10.3390/children10040680

**Published:** 2023-04-03

**Authors:** Faten Sahli, Hajer Sahli, Omar Trabelsi, Nidhal Jebabli, Makram Zghibi, Monoem Haddad

**Affiliations:** 1 Higher Institute of Sport and Physical Education of Ksar-Said, University of Manouba, Manouba 2010, Tunisia; 2 Research Unit: Sportive Performance and Physical Rehabilitation, High Institute of Sports and Physical Education of Kef, University of Jendouba, Kef 7100, Tunisia; 3 Research Unit: Physical Activity, Sport, and Health, UR18JS01, National Observatory of Sport, Tunis 1003, Tunisia; 4 Physical Education Department, College of Education, Qatar University, Doha 2713, Qatar

**Keywords:** physical education, extrinsic motivation, secondary students, peer-assisted learning

## Abstract

Objective: This study aimed at assessing the effects of two verbal encouragement modalities on the different offensive and defensive performance indicators in handball small-sided games practiced in physical education settings. Methods: A total of 14 untrained secondary school male students, aged 17 to 18, took part in a three-session practical intervention. Students were divided into two teams of seven players (four field players, a goalkeeper, and two substitutes). During each experimental session, each team played one 8 min period under teacher verbal encouragement (TeacherEN) and another under peer verbal encouragement (PeerEN). All sessions were videotaped for later analysis using a specific grid focusing on the balls played, balls won, balls lost, shots on goal, goals scored, as well as the ball conservation index (BCI), and the defensive efficiency index (DEI). Results: The findings showed no significant differences in favor of TeacherEN in all the performance indicators that were measured, whereas significant differences in favor of PeerEN were observed in balls played and shots on goal. Conclusions: When implemented in handball small-sided games, peer verbal encouragement can produce greater positive effects than teacher verbal encouragement in terms of offensive performance.

## 1. Introduction

Effective teaching in physical education (PE) does not only rely on planning and implementing the best evidence-based didactic materials and pedagogical strategies to ensure students are making progress in their motor learning [1,2]. There are times when PE teachers are required to address other intangible parameters in order to increase their students’ motivation and, in turn, deeply engage them in the learning process [3].

PE contributes to the social growth of each student and exposes them to a variety of rules and practices essential to the acquisition of the imperative knowledge of “living together” [4]. In other words, PE classes are widely recognized as a hot bed of socio-constructivist learning activities which emphasize the role of social interaction in the process of knowledge acquisition [5,6,7]. Teaching team sports during PE classes in school settings is not merely a matter of honing students’ motor, technical, and tactical skills [8]. Students must be confronted with situations that foster reflective practices, engaging them in a process of socio-constructivist learning, and thus, continuous adaptation [7,9].

In the last decade, it has been well documented that the different kinds of verbalizations that a teacher uses during classroom interactions (e.g., instruction, encouragement, feedback, etc.) can be beneficial for both the teacher’s practices and students’ learning in PE [7,9,10]. The PE class is an opportunity to be assisted by others (teacher or peers) in order to perform a technical skill, to apply the tactics of a given sport and to seek solutions to the problems encountered [11]. The opportunity to work under the encouragement of the teacher is particularly important in order to analyze and understand the teaching-learning process [12,13]. Indeed, verbal encouragement can enhance students’ motivation, which is the driving force of their actions, desires, and needs [14]

In handball, teamwork is an essential component of athletic performance [15,16]. Team games/sports consist of a chain of problems to be solved on the way to achieving a successful collective outcome, often occurring in a random order [17]. Verbal encouragement, either of the teacher or peers, has previously been shown to be a major contributor to tackling those problems and therefore enhancing learning in team sports during PE classes [7,10,17]. Indeed, team sports involve mental, emotional, and affective demands, which can play a vital role in the success of motor, tactical, and technical performance during training sessions and competitions [18]. Those sports regularly involve situations of confrontation between two fairly balanced teams [19]. While being involved in such situations, students from both teams may benefit from extrinsic motivation to overcome the difficulties encountered as a result of confrontation [7,11,20,21].

Handball is considered a highly demanding team sport, featuring high-intensity activities and requiring specific technical competencies, tactical awareness, and physical exertions such as running, sprinting, and jumping, as well as regular throwing, blocking, and jostling between players [22]. All those technical, tactical, and physical qualities are essential to achieving good defensive and offensive performance indicators during games; such indicators include the number of the balls played, lost, won, as well as the number of shots on goal and the goals scored [23]. For example, a high number of shots on goal achieved by a team during a single handball game indicates a good offensive performance. This sport raises the problem of group dynamics: two teams facing off on the basis of a set of rules, all while experiencing conflicts of interest and chasing individual and collective goals [24,25]. The interests of one player (student) do not necessarily have to be aligned with the group’s interest. Therefore, it is crucial for the students to be active in terms of intra-group communication and for the teacher to intervene, often in the form of encouragement and instructions, to motivate the students and persuade them to go further in their learning journey [26]. Indeed, the findings of Sahli et al. [27] showed that positive effects of combined verbal encouragement and technical instruction during handball small-sided games on students’ passing efficiency can be observed during PE classes.

Within the framework of our research and based upon the literature reviewed, several questions have been raised. We pondered whether peer verbal encouragement can produce greater positive effects than teacher verbal encouragement in terms of offensive and defensive performances in handball small-sided games. Specifically, to what extent can each of these encouragement modalities influence performance indicators such as balls played (passing efficiency), balls lost, balls won, shots on goal, and goals scored? We hypothesized that peer verbal encouragement would be more effective than teacher verbal encouragement in enhancing all performance indicators in handball small-sided games. In brief, this study mainly aimed at comparing the effects of two verbal encouragement modalities (teacher vs. peer verbal encouragement) on secondary school students’ offensive and defensive performance in handball small-sided games during PE classes.

## 2. Materials and Methods

### 2.1. Study Design

The experimental protocol of this study consists in teaching a handball learning unit with verbal encouragement from the teacher (TeacherEN) or peers (PeerEN) in a boys-only class. The main learning situation was composed of two 8 min periods of play between two teams of students in a reduced half-court playground (dimensions: 20 m long/10 m wide).

The selection of the population depended on decisions taken following discussions with the administration of the secondary school that agreed to host the experiments. It was a school of about 450 students, was located in a Tunisian coastal area, was somewhat unprivileged, and was socially homogeneous due to mainly enrolling students from lower classes. Our study included 14 untrained secondary school male students, aged 17.36 ± 0.49, in their third year of the Tunisian secondary education system (Table 1). Students were deemed eligible for inclusion in the study if they met the following criteria: (a) the student must not have previous experience in competitive handball, and (b) must be a healthy individual, physically capable of engaging in high-intensity exercise. Parents/guardians of the students received an informed consent letter containing detailed information about the study procedures. By signing the letter, they approved the participation of their dependents in the study.

Students completed three handball learning sessions over the course of three successive weeks, with the rate of one session per week. During each session, students were required to play two 5 × 5 small-sided handball games, each lasting 8 min. The duration of handball SSGs was set based on recommendations of Corvino et al. [28]. The 14-student class was divided by the teacher into two equal teams of 7 students: each team was composed of 4 players, a goalkeeper and 2 substitutes. It should be noted that substitutions were made by the players themselves and at any time over the course of the game. Each team plays twice for an 8 min period, once under the verbal encouragement of the two substitute players, and another under the verbal encouragement of their teacher. During the first 8 min play period of each session, the teacher intervened verbally to encourage players of team A, while the substitute players of team B intervened, as well to encourage their teammates. Roles were reversed during the second 8 min play period: the same encouragement pattern as that of the first period was maintained, only this time, the teacher encouraged team B while team A was encouraged by the substitute players. Verbal encouragement expressions were standardized across conditions (TeacherEN and PeerEN). The teacher and the peers used the following expressions for encouragement: “Go, well done, everything is fine, this is great, don’t give up, great, courage, go ahead, try again, come on, you will get there, trust yourself, and you can”. Verbal encouragement has been recognized as a potential confounder in experimental research in the field of sports and physical activity [29]. Standardization of encouragement in within-subject research designs should reduce confounding, especially where there is potential for experimenter expectancy bias. Standardization should also increase test–retest reliability in non-research contexts [29].

The teacher who participated in the study was a 40-year-old male PE teacher, holding an undergraduate degree in sports science and a level-three coaching license in handball. He had 15 years of teaching experience in secondary education, which made him a qualified specialist in his discipline. His high-level of personal experience was further acclaimed by his peers and pedagogical inspectors. 

Using a GoPro HERO4 camera, all experimental sessions were recorded for later analysis. The investigation tool used to study the effects of either teacher or peer verbal encouragement on the different learning indicators in handball was an analysis grid, initially developed by Gréhaigne [30], and specifically designed to analyze performances in team sports. This observation grid was previously used by Zerai [15] to study the effects of learning with peer verbalizations in handball games on the decision-making process in adolescent girls. After a first observation session, Zerai [15] verified the consistency between data collected through live observation and that recorded through video observation. Due to the game’s slow pace, there were hardly any consistency errors, making it easy to observe. The grid allowed the collection of the following data: (a) balls played, (b) balls lost, (c) balls won, (d) shots on goal, and (e) goals scored during each 8 min play period. Offensive and defensive indexes were also considered for analysis.

- Balls played: this indicator reports on ball possession through statistics of successfully passed/exchanged balls;
- Balls won: this indicator provides details about the efficiency of a team in gaining possession of the ball (interception) from the opposing players;
- Balls lost: this indicator reports on the rate of ball loss due to either a violation or an interception of the ball by the opposing team;
- Shots on goal: an indicator of score. A shot on goal is a shot that is on net. The outcome of a shot on goal must be either a save by the goalkeeper or the defending team or a goal by the attacking team;
- Goals scored: this is an indicator of offensive efficacy. Each goal a team scores leads to an increase in the offensive efficiency;
- Ball Conservation Index (BCI = balls lost/balls played): This index varies from 0 (when no balls are lost) to 1 (when the number of balls lost is equal to the number of balls played). For scores between 0.5 and 0.8, the relationship between these two values highlights a team’s ability to retain the ball in a given opposition situation [31];
- Defensive Efficiency Index (DEI = balls won/balls lost): This index studies the ratio of balls won vis-à-vis with balls lost in order to emphasize the team’s efficiency in retrieving and then conserving the ball as a result of a better offensive and defensive organization. This index varies from 0 (when no balls are conquered) to 1 (when the number of balls won is equal to the number of balls lost). It can also exceed the threshold of 1 if the number of balls won is greater than the number of balls lost [32].

### 2.2. Statistical Analysis

Data were presented as means and standard deviations (SDs) in text and figures. After confirming that most of the datasets are not normally distributed using the Kolmogorov Smirnov test, we performed the Wilcoxon signed-rank test to compare data collected under peer and teacher encouragement for each group during all three scheduled practical sessions. Statistical significance was accepted at *p* < 0.05. Data were analyzed using the SPSS 22 package (SPSS Inc., Chicago, IL, USA).

## 3. Results

### 3.1. Technical Performance

As shown in Table 2, no significant differences in favor of the TeacherEN modality were identified across all three sessions in all performance indicators. On the other hand, the number of balls played was mostly greater (at *p* < 0.05) when the students of both teams played under PeerEN. Significant differences in favor of PeerEN were also observed in the number of shots on goal achieved by team A during session 1 (at *p* < 0.05) and team B during session (at *p* < 0.05).

### 3.2. Tactical Performance

#### 3.2.1. Ball Conservation Index (BCI)

**Figure 1:**
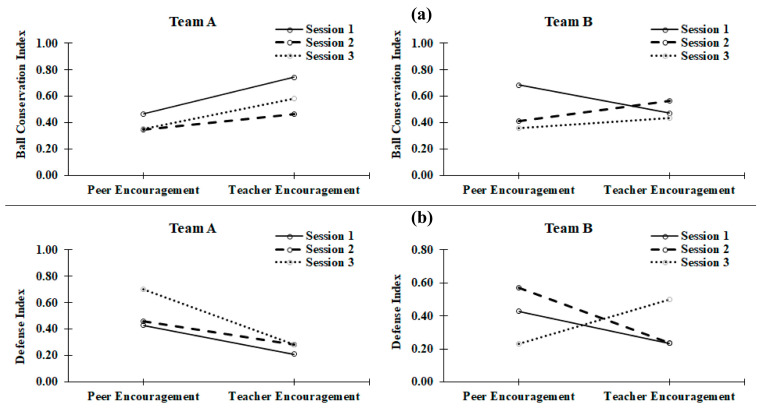
(**a**) The effects of two different modalities of verbal encouragement on ball conservation index (BCI); (**b**) the effects of two different modalities of verbal encouragement on defensive efficiency index (DEI).

Figure 1 shows that during all three sessions, the BCI was better in team A under PeerEN than under TeacherEN, with no significant difference. The index was equal to 0.47 during the first session, 0.52 during the second, and 0.34 during the third, whereas when the students switched to TeacherEN, the indices became higher: 0.73; 0.58, and 0.58, respectively.

Figure 1 also illustrates that during the second and third session, the BCI was in favor of PeerEN in team B (Session 2: 0.70 vs. 0.85; session 3: 0.50 vs. 0.64). On the other hand, during the first session, this index was lower under PeerEN (0.58) than under TeacherEN (0.34).

#### 3.2.2. Defensive Efficiency Index (DEI)

For team A, we noticed a non-significant increase in favor of PeerEN during the three sessions (0.43, 0.46, and 0.70, respectively) compared to TeacherEN (0.21, 0.29, and 0.29, respectively).

For team B, this DEI was non-significantly better during under TeacherEN during the first session (0.70 vs. 0.29) and during the third (0.50 vs. 0.23). The DEI became in favor of PeerEN during the second session (0.46 vs. 0.29).

Despite these differences, which were often in favor of PeerEN, statistically significant differences between the two verbal encouragement modalities in DEI did not exist across all three sessions according to the non-parametric Wilcoxon test (see Figure 1).

## 4. Discussion

This study mainly aimed at comparing the effects of two verbal encouragement modalities (teacher vs. peer verbal encouragement) on secondary school students’ offensive and defensive performance in handball small-sided games during PE classes. The results of our investigations showed that, during all of the handball small-sided games carried out as part of the three-session practical intervention in physical education (PE) settings, no significant differences in all performance indicators were recorded in favor of the TeacherEN. However, such differences were observed in offensive indicators such as balls played and shots on goal when the students played under PeerEN. Even when statistical significance was not confirmed, values recorded under PeerEN exceeded those recorded under TeacherEN at most times. These initial findings advocate for the importance of promoting peer-assisted learning situations while teaching handball in PE classes, during which, students are given the freedom to verbally encourage one another within the framework of an active learning pedagogy. In the Tunisian context of PE, the student decision-making process leans heavily on the relationship that bonds him/her to the sport being taught by the teacher [33]. Strengthening this relationship while teaching team sports often requires the engagement of, not only the teacher, but also the peers in the process of providing sources of extrinsic motivation, such as verbal encouragement, to students engaging in a given motor, technical, or tactical learning task [17,27].

In all three practical sessions scheduled as part of the experiment, most of the offensive indicators measured during the handball small-sided games (i.e., balls played and shots on goal) were significantly greater when the students played under PeerEN. These findings could be attributed to improvements in students’ cognitive functioning, particularly auditory and visual information processing, which may have resulted from peer verbal encouragement being an external factor driving extrinsic motivation during the handball small-sided games. Indeed, extrinsic motivation is likely to play a significant role during the different stages of information processing by the human brain [34,35]. In other words, external motivational stimuli (e.g., peer verbal encouragement) can focus the attention of the player on collecting and processing information (e.g., opponents’ collective and individual movement, ball flow, etc.) useful for the elaboration and implementation of effective performance strategies [36]. This pattern of results could also be explained by an enhanced understanding of the game as well as enhanced offensive organization.

The offensive and defensive organizations tended to be more effective when students played under the verbal encouragement of their peers; however, this was not statistically confirmed. These findings lead us to say that the team performance as a whole improved as a result of PeerEN. The difference in the number of balls successfully played indicates a greater volume of ball possession and provides insight into the efficient exchange of the ball between teammates. As students played under verbal encouragement from their teammates (substitute players), they were found to be more adept at solving cascades of problems encountered during the handball small-sided game. Innes et al. [37] have shown that peer verbal encouragement improves self-efficacy and physical performance, particularly in children.

Through analyzing the ball conservation indexes (BCI), as well as the defensive efficiency indexes (DEI) under the two different modalities of verbal encouragement (TeacherEN and PeerEN), we concluded that ball conservation and defensive efficiency improved whenever students played under PeerEN, still the differences were not statistically significant.

The findings reported in this study suggest that there is a significant difference in favor of peer verbal encouragement on some offensive performance indicators of secondary school students in handball small-sided games. These results can be explained by the fact that students feel freer and more spontaneous with verbal encouragement provided by their teammates. In the same line, Zghibi et al. [5] showed that when students interact in the absence of their teacher, they manage to provide richer and more cogent answers in response to their peers’ questions. Moreover, in the absence of the teacher, the students exchange verbally with much spontaneity and more discursive freedom. On the other hand, when students converse in the presence of their teacher, the discourse becomes less constructive and less conflictual, making answers to the questions posed by the teacher less argumentative [38]. Indeed, studies conducted in the same context call upon teachers to provide a space of freedom for students to interact independently of the teacher’s intervention [38]. Potential theoretical and pedagogical implications of external motivation are drawn to enhance the quality and effectiveness of external motivation. Furthermore, encouraging expressions, such as VE, are thought to be a suitable and powered factor not only to induce emotional states, but also to subsequently affect performance or cognitive processing [39].

Overall, PeerEN often resulted in better performance indicators during the small-sided games conducted within the framework of this study, particularly offensive indicators. Disappointingly, the differences between all defensive indicators collected under PeerEN and TeacherEN were not strong enough to produce a statistical significance. This could be due to the reduced time allotted for each play period (8 min) and possibly due to restrictions imposed on the times substitutes were allowed to verbally encourage their peers (three times per play period). Another drawback of the methodology implemented in this work is the absence of a control group. In fact, a group of subjects practicing handball small-sided games under normal conditions, where no verbal encouragement is involved, could have made it easier to interpret the results of each modality of verbal encouragement and conduct more accurate comparisons. Further replication and verification of these research results are needed, as we strongly believe that a larger sample, a longer game duration, and an increased amount of peer encouragement can lead to a different pattern of results and stronger statistical power.

## 5. Conclusions

This study is innovative not only in terms of research in didactics and pedagogy of physical education, but also in terms of research in instructional science in general, as it suggests a new teaching model based on a coupling between peer and teacher verbal encouragement. During handball small-sided games played under peer verbal encouragement, offensive efficiency improved. Despite the low statistical significance, it is legitimate to conclude that, compared to teacher verbal encouragement, peer verbal encouragement increased the number of balls played and shots on goal in major cases. In light of these conclusions, teachers and coaches are called upon to consider peer verbal encouragement, alongside their own encouragement, as a pedagogical tool during handball learning sequences. Future research should consider investigating the effects of verbal encouragement on cognitive and psychological responses of children while practicing individual sports during physical education classes. 

## Figures and Tables

**Table 1:** Demographic and anthropometric characteristics of participants.

Gender	Males	14
	Females	0
Age	17 years	9
	18 years	5
Mean ± SD of years of experience in handball	0 years	
Mean ± SD Height	171.74 ± 3.18 cm	
Mean ± SD Weight	67.81 ± 2.47 kg	

**Table 2:** The effects of two different modalities of verbal encouragement on technical performance.

		Team A				Team B			
		Peer Encouragement	Teacher Encouragement	*p* Value		Peer Encouragement	Teacher Encouragement	*p* Value	
				Z	Sig.			Z	Sig.
Session 1	Balls played	30	26	−0.55	0.581	30	25	−2.06	0.039 *
	Balls won	6	4	−0.82	0.414	6	4	−1.00	0.317
	Balls lost	14	19	−1.13	0.257	14	17	−1.34	0.18
	Shots on goal	10	6	−1.99	0.046 *	12	9	−1.00	0.317
	Goals scored	3	3	0.00	1.000	3	3	−0.58	0.564
Session 2	Balls played	38	30	−2.08	0.037 *	34	30	−1.98	0.046 *
	Balls won	6	4	−1.00	0.317	8	4	−1.73	0.083
	Balls lost	13	14	−1.60	0.109	14	17	−1.73	0.083
	Shots on goal	7	5	−1.89	0.059	6	4	−1.41	0.157
	Goals scored	3	2	−1.00	0.317	5	2	−1.73	0.083
Session 3	Balls played	29	24	−1.89	0.059	36	32	−2.00	0.046 *
	Balls won	7	4	−1.63	0.102	3	7	−1.00	0.317
	Balls lost	10	14	−1.13	0.257	13	14	−1.00	0.317
	Shots on goal	7	2	−1.34	0.180	8	4	−2.00	0.046 *
	Goals scored	4	1	−1.73	0.083	2	2	−1.00	0.317

* *p* value < 0.05.

## Data Availability

Not applicable.
